# Physical literacy assessment in adults: A systematic review

**DOI:** 10.1371/journal.pone.0288541

**Published:** 2023-07-14

**Authors:** Aia Boldovskaia, Nuno Manuel Gonçalves Dias, Marlene N. Silva, Eliana V. Carraça

**Affiliations:** 1 Faculdade de Educação Física e Desporto, Universidade Lusófona de Humanidades e Tecnologias, Lisboa, Portugal; 2 Programa Nacional de Promoção da Atividade Física, Direcção-Geral da Saúde, Lisboa, Portugal; Abo Akademi University Faculty of Economics and Social Sciences: Abo Akademi Yhteiskuntatieteiden ja kauppatieteiden tiedekunta, FINLAND

## Abstract

Physical literacy is a multidimensional construct that has been defined and interpreted in various ways, one of the most common being “the motivation, confidence, physical competence, knowledge and understanding to maintain physical activity throughout the life course”. Although its improvement can positively affect many behavioral, psychological, social, and physical variables, debate remains over an appropriate method of collecting empirical physical literacy data. This systematic review sought to identify and critically evaluate all primary studies (published and unpublished, regardless of design or language) that assessed physical literacy in adults or have proposed measurement criteria. Relevant studies were identified by searching four databases (Pubmed, SportDiscus, APA PsycINFO, Web of Science), scanning reference lists of included articles, and manual cross-referencing of bibliographies cited in prior reviews. The final search was concluded on July 15, 2022. Thirty-one studies, published from 2016 to 2022, were analyzed. We found seven instruments measuring physical literacy in adults, of which six were questionnaires. The Perceived Physical Literacy Instrument was the first developed for adults and the most adopted. The included studies approached physical literacy definition in two ways: by pre-defining domains and assessing them discretely (through pre-validated or self-constructed instruments) and by defining domains as sub-scales after factorial analyses. We found a fair use of objective and subjective measures to assess different domains. The wide use of instruments developed for other purposes in combined assessments suggests the need for further instrument development and the potential oversimplification of the holistic concept, which may not result in a better understanding of physical literacy. Quality and usability characteristics of measurements were generally insufficiently reported. This lack of data makes it impossible to compare and make robust conclusions. We could not identify if any of the existing physical literacy assessments for adults is appropriate for large-scale/epidemiological studies.

## Introduction

Physical literacy is a multidimensional construct that has been defined and interpreted in various ways, one of the most common being “the motivation, confidence, physical competence, knowledge and understanding to maintain physical activity throughout the life course” [1, 2]. Its improvement can positively affect many behavioral, psychological, social, and physical variables [1], but does it have the power to reduce the burden of non-communicable diseases and boost well-being for life? By definition physical literacy is a gateway to lifelong participation in physical activity, with its benefits being well established [3]. Some authors consider physical literacy a logical and fundamental determinant of health—through its formative role in shaping lifelong trajectories—and call for studies to validate this relationship [4, 5].

Despite its widely recognized value, uncertainty around the concept prevails [6]. The lack of clarity, marked by a variety of definitions and interpretations adopted globally [7], undermines its operationalization [8]. Developing public health strategies, policies and guidelines, as well as intervention programs, requires a clear understanding of what components constitute physical literacy, and how it can be observed and assessed. Debate remains over an appropriate method of collecting empirical physical literacy data and even the (im)possibility of such measurement [9].

Physical literacy encompasses a cluster of domains conceptually linked together. Domains are defined differently within each distinct physical literacy interpretation, commonly including affective, physical, cognitive, behavioral, and social domains [10–12]. Most existing instruments assess attributes of physical literacy under either one or two domains while marginalizing the rest [9], and this approach may diminish the holistic intent and philosophical underpinnings of the concept [6]. Another issue is whether physical literacy should be qualified, interpreted, and judged against pre-established benchmarks and standards. For instance, Margaret Whitehead argued for comparisons to be made with one’s previous assessment and never in relation to others [13].

Physical literacy is philosophically founded on respecting the nature of the human being and considers every individual without concern of age group or living place, but practically all existing assessment instruments were developed against this contention [9], being focused on children [11]. Given its potential public health contribution, we need to understand how to access, maintain and enhance physical literacy in adults.

In 2018, Edwards made the first and the most embracing effort so far to systematize physical literacy and related constructs assessments in different age groups [11]. It seems that Edwards’ work made a significant impact, as during the foundational phase of the current research we have found a recent increase in studies concerning physical literacy in adults. Since then, four more studies reviewed physical literacy assessments in adults. The first was limited to the physical domain assessments in older adults [14]. The second was focused on definitions and constructs and was limited to aging adults [15]. The third only included explicit assessment instruments and evaluation tools, being able to identify two for adults [9]. The authors of the fourth didn’t find any validated measurement to access physical literacy in adults and have chosen to include measurements “useful for measuring the different elements of the three overall domains of physical literacy” in their self-reported measurements review [16]. All mentioned reviews were limited to peer-reviewed literature (except for Petrusevski’s) published in English.

To systematize recent progress, we sought to identify all studies that measured physical literacy in adults or have proposed measurement criteria. To our knowledge, this is the first systematic review to consider published and non-published literature, without language restriction, concerning physical literacy assessment instruments and attempts in adults. Therefore, the purpose of the current paper is: 1) To systematically review and compare existing physical literacy assessment attempts in adults in relation to its: (a) alignment to the physical literacy concept; (b) measurement properties; and 2) To propose an appropriate instrument to evaluate physical literacy in adults that can contribute to public health promotion.

## Methods

This systematic review followed the Preferred Reporting Items for Systematic Reviews and Meta-Analyses (PRISMA) [17]. We developed a search strategy in advance to identify related literature. The protocol information was submitted for registration in the International Prospective Register of Systematic Reviews (PROSPERO) (registration number CRD42022340204).

### Eligibility criteria

Considering the scarcity of existing evidence, this systematic review sought to identify all studies (observational or experimental) that measured physical literacy in adults (age of 18 or older), using a physical literacy assessment method (qualitative or quantitative), or that have proposed measurement criteria. All primary studies, regardless of design, were considered eligible for inclusion to identify potentially relevant studies. Published (peer-reviewed) and unpublished literature, in the form of journal articles, dissertations/theses, pre-prints or conference papers, were examined. No language restrictions were applied to maintain the inclusive character of this systematic review and avoid language bias [18]. Studies were excluded if they included samples of children or teenagers. Books or book chapters, review articles, commentaries, meta-analyses, editorials, protocol papers, conference abstracts, and systematic reviews, were excluded. Dissertations and theses which resulted in published journal articles were excluded to avoid duplication.

### Information sources

Relevant studies were identified by searching four databases (Pubmed, SportDiscus, APA PsycINFO, Web of Science), scanning reference lists of included articles, and manual cross-referencing of bibliographies cited in prior reviews [9, 11, 14, 15]. Google Scholar was used as a supplement search tool to identify grey literature not included in library databases (e.g., theses and dissertations). Each of the databases was searched independently.

### Search strategy

Searches included combinations of three sets of terms following PICOS: (a) terms concerning the population of interest (e.g., adults), (b) terms concerning type of “intervention”/exposure of interest, which in this case was related to measurement issues (e.g., assessment, questionnaire, instrument), and (c) terms concerning the outcomes of interest (physical literacy). All types of study design (i.e., observational, and experimental) were included, thus there were no restrictions on study design or comparator. A full search example can be seen in Table 1. Publication date restrictions were not applied in any search. The initial search was concluded on May 12, 2022, and the final on July 15, 2022, before data extraction.

**Table 1:** Full search example.

#1	Adults OR Students
#2	Assessment OR Measurement OR Test OR Tool OR Instrument OR Battery OR Method OR Observation OR Indicator OR Evaluation OR Questionnaire
#3	“Physical literacy”
#	1 AND 3
#	1 AND 2 AND 3

### Selection process

**Fig 1:**
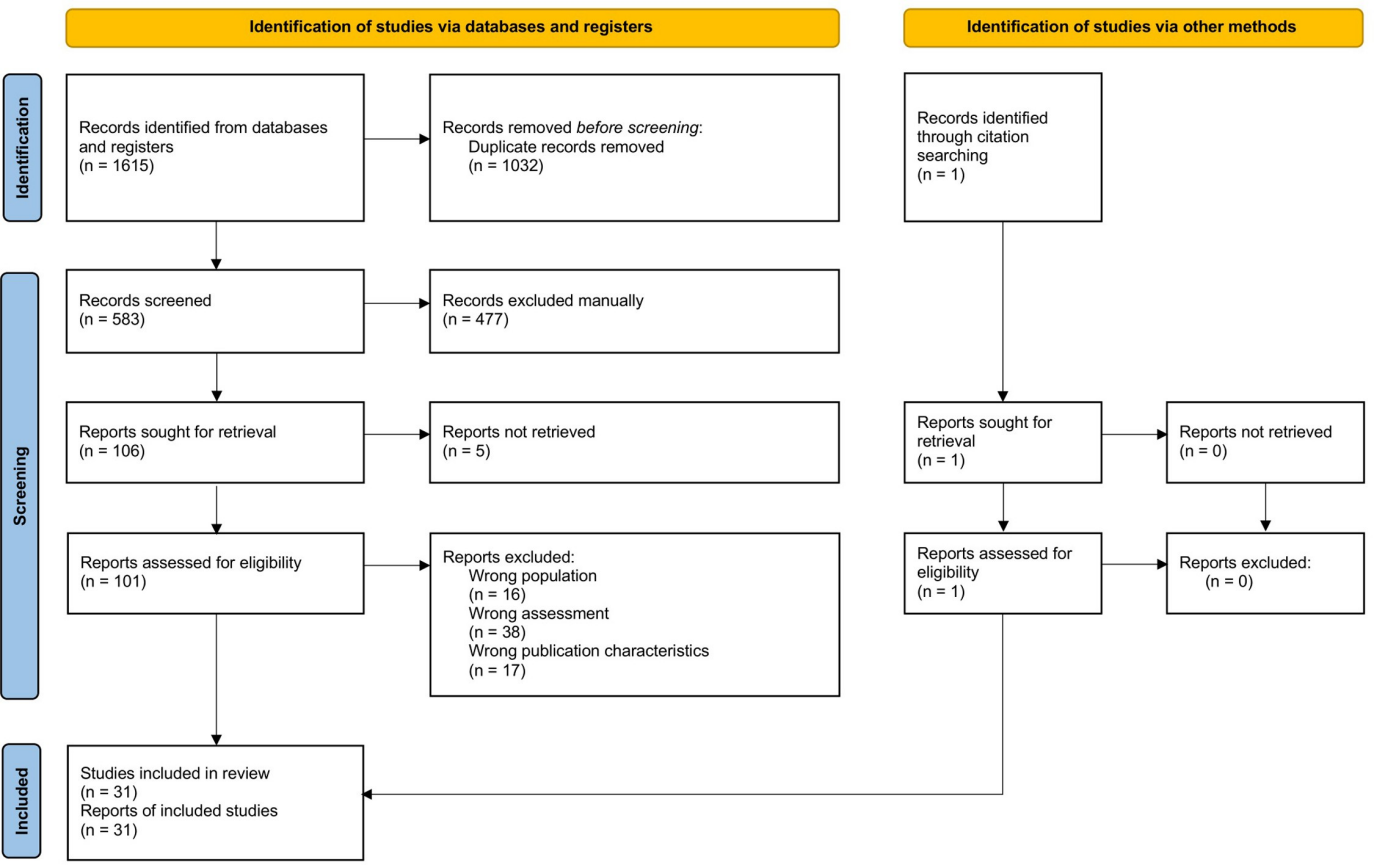
PRISMA flow diagram for the identification of the included studies.

The study selection process is detailed in the PRISMA flow chart (Fig 1). Following the initial search, all records were exported to Cadima software [19] for screening. Duplicates were manually eliminated. Titles, abstracts, and full texts of relevant studies were screened by two authors to identify studies that fulfilled the eligibility criteria. When the full text was not accessible, we contacted the studies’ corresponding authors via ResearchGate or email. Results were cross-checked. Decisions to include or exclude studies in the review were made by consensus. Reference lists of identified articles and prior reviews were screened to ensure that no relevant studies were overlooked.

### Data extraction process, data items and synthesis methods

A data extraction form was developed, informed by the PRISMA statement [17]. Data extraction was performed by two authors independently and included information about participants’ characteristics, adopted physical literacy definition, assessment content, assessment characteristics, and measurement properties. A complete list of extracted items is provided in S1 File. Missing information was coded as not reported. Discrepancies during the extraction process were discussed with a third author and solved by consensus.

Following the example of Rockliffe, Google translate was initially used to assess the eligibility of non-English language abstracts and full texts [20]. Studies deemed eligible were sent for data extraction to volunteer translators identified through personal contacts.

Selected studies were evaluated and analyzed. The retrieved data were organized into a table to facilitate the synthesis process. The full data sheet is available in S2 File. A qualitative synthesis was carried out using descriptive analysis and constant comparison method to uncover themes connected to physical literacy assessment in adults [21, 22]. The analysis method included three stages: 1) Summarizing the included studies’ characteristics; 2) Summarizing extracted data by identifying patterns, parallels, or correlations and grouping data into themes; 3) Organizing results in illustrative extracts and analytic narrative. More specifically, the data was first coded with descriptive tags (e.g. “original assessment”, “physical domain”, “young adults”, “five domains”), then tags were compared to identify categories, similarities, and differences. Tags were refined in each iteration to establish categories (e.g. “young adults”, “adults”, “older adults”), and identify patterns (e.g. “practical task” was only used to access physical attributes). Studies were summarized based on identified categories and an analytic narrative was developed based on identified patterns.

To assess the quality of the available physical literacy assessment instruments, we retrieved psychometric properties and feasibility data from the studies. These data included measures of reliability, validity, and other relevant psychometric properties, such as factor structure, and item response characteristics. Feasibility data were also collected, including information on staff and equipment required, assessment time, and other available details.

### Study risk of bias assessment

Two authors independently assessed the quality of each study using the Effective Public Health Practice Project Quality Assessment Tool (EPHPP) [23]. First, each study was marked as “strong,” “moderate,” or “weak” in eight categories following EPHPP assessment sheet: selection bias (representativeness), study design, confounders, blinding, data collection methods, withdrawals and dropouts, intervention integrity, and analysis. The overall global rating was then determined: “strong” for studies with no weak ratings, “moderate” for studies with one weak ranking, and “weak” for studies with two or more weak ratings. Discrepancies were discussed with a third author, and the final grade decision was made by consensus. Quality appraisal summary is available in S3 File.

## Results

The search strategy resulted in 1616 studies, and 102 were eligible for full-text screening. Characteristics of 31 studies included in the current review are summarized in Table 2. Most studies that attempted to measure physical literacy in adults were published after 2020. Studies were conducted in nine different countries—half of them in China—with only three published in non-English languages. Half of the studies assessed physical literacy in young adults in university settings, while older adults were underrepresented. The full raw data is available in S2 File.

**Table 2:** Studies summary.

Characteristic	Outcome (number of studies)
**Year**	2016 (1) [24], 2017 (1) [25], 2018 (1) [26], 2019 (2) [27, 28], 2020 (11) [29–39], 2021 (7) [40–46], 2022 (8) [47–54]
**Language**	English (28), Czech (1), Portuguese (1), Turkish (1)
**Country**	China (17), Canada (4), USA (3), Austria (2), Turkey (2), Czech Republic (1), Denmark (1), Malaysia (1), Portugal (1)
**Publication type**	Journal article (27), thesis (3), conference paper (1)
**Age group**	Young adults (17), adults (11), older adults (3)
**Setting**	University/college (17), school (4), community (4), professional development program (2), independent living/day care center (2), childhood education center (1), general public (1)
**Physical literacy assessment approach**	Pre-validated assessment (12), combined (7), combined with composite score (4), adaptation of pre-validated assessments (4), original assessment (4)
**Type of measurements employed**	Questionnaire (29), practical task (5), objective measure (5), interview (1)

We classified assessment approaches into five categories: 1) pre-validated physical literacy assessments; 2) combined assessments of pre-validated or self-constructed scales without a composite score; 3) combined assessments of pre-validated or self-constructed scales with a composite score; 4) adaptations of pre-validated physical literacy assessments; 5) original assessments. Original assessments included two survey-based instruments (Perceived Physical Literacy Instrument, College Student Physical Literacy Questionnaire), a speech database for assessing physical competence under the concept of physical literacy, and a focus group to evaluate confidence, physical competence, sense of self, and knowledge in relation to physical literacy. Almost all studies utilized at least one questionnaire.

Physical literacy was defined consistently within included studies, mostly using slightly different versions of Margaret Whitehead’s definitions. All adopted definitions and references—as provided by authors—are listed in Table 3.

**Table 3:** Physical literacy definitions.

Definition	Number of studies
Motivation, confidence, physical competence, knowledge and understanding to value and take responsibility for engagement in physical activities for life [2, 55–60]	24
The motivation, confidence, physical competence, understanding and knowledge to maintain physical activity at an individually appropriate level throughout life [60]	2
The development of motivation, confidence, physical competence, knowledge, and understanding to value and engage in a wide variety of physical activities and environments that benefit the person as a whole [57]	1
An individual’s prerequisites to participate in and adhere to physical activities throughout the life-course [2]	1
A concept that values physical activity for the individual’s health and active living style throughout the life course [61]	1
The ability to move with competence and confidence in a wide variety of physical activities in multiple environments that benefit the health development of the whole person [62]	1
Daily behavior, knowledge, self-efficacy, and motivation to be physically active throughout the lifetime [2]	1

Explicit physical literacy instruments—six questionaries and one objective measure—are summarized in Table 4. Available psychometric properties of the instruments, as well as feasibility characteristics, are reported in Table 5.

**Table 4:** Explicit physical literacy instruments.

Instrument (number of studies)	Type	Physical literacy domains assessed	Target population
PPLI (9)	Questionnaire, 9 items	Self and self-confidence, self-expression and communication with others, knowledge and understanding	Young adults, adults, older adults
PPLI-SC (4)	Questionnaire, 8 items	Motivation, confidence and physical competence, interaction with the environment	Young adults
PPLI (turkish) (2)	Questionnaire, 9 items	Knowledge and understanding, sense of self and self-confidence, communication	Adults
SPPLI (1)	Questionnaire, 11 items	Attitude toward physical activity, physical activity ability, sociality around physical activity	Older adults
DSPG (1)	Questionnaire, 22 items	Confidence, self-efficacy, relative ranking of literacies, physical competence	Young adults
CSPLQ (1)	Questionnaire, 38 items	Physical and behavioral, cognitive, emotional	Young adults
Speech database (1)	Objective measure	Physical competence	Young adults

PPLI–Perceived Physical Literacy Instrument

PPLI-SC–Perceived Physical Literacy Instrument Simple Chinese

SPPLI–Senior Perceived Physical Literacy Instrument

DSPG–*Dotazníku sebehodnocení pohybové gramotnosti*

CSPLQ—College Student Physical Literacy Questionnaire

**Table 5:** Physical literacy instruments validity and feasibility.

Instrument and model	Validity and reliability reported	Feasibility
PPLI 9 tems, 3 factors	**Internal consistency** Full scale Cronbach’s α 0.82 [24] Domains Cronbach’s α 0.73–0.76 [24], 0.66–0.93 [40], ≥0.85 [36] **Model fit** Chi-square (p>0.05), CFI = 0.95, RMSEA = 0.038 [24]	Online/on-site application, 8–10 minutes required for assessment
PPLI-SC 8 items, 3 factors	**Internal consistency** Full scale Cronbach’s α 0.91 [42] Domains Cronbach’s α 0.79–0.83 [30] **Model fit** RMSEA 0.03, AGFI 0.96, Normed Chi-square 1.32, NFI 0.97, CFI 0.99, TLI 0.99, PNFI 0.59 [30]	Online/on-site application, trained assistants and 10 minutes required for assessment
PPLI (13urkish) 9 items, 3 factors	**Internal consistency** Full scale Cronbach’s α 0.81 [32], 0.88 [44] Domains Cronbach’s α 0.71–0.87 [44] **Model fit** Normed Chi-square 1.94, RMSEA 0.046, SRMR 0.084. RMR 0.27, NFI 0.92, NNFI 0.93, CFI 0.94, GFI 0.96, AGFI 0.97 [32]	Online/on-site application
SPPLI 11 items, 3 factors	**Internal consistency** Full scale Cronbach’s α 0.90 [48] Domains Cronbach’s α 0.80 to 0.90 [48]	Trained assistants required
DSPG 22 items, 4 factors	**Internal consistency** Full scale Cronbach’s α 0.72 [34] **Temporal stability** (1 month) Intraclass correlation coefficient 0.85 [34]	Not reported
CSPLQ 38 items, 3 factors	**Internal consistency** Full scale Cronbach’s α 0.961 [49] Domains Cronbach’s α 0.900–0.936 [49] **Concurrent validity** (athletic ability, physical condition, physical attractiveness, physical fitness, frequency of physical activity, and length of physical activity) *p* < 0.05 [49]	Online application, 10–15 minutes required for assessment
Speech database	Not applicable [50]	On-site application, recording equipment and trained assistants are required.

PPLI–Perceived Physical Literacy Instrument

PPLI-SC–Perceived Physical Literacy Instrument Simple Chinese

SPPLI–Senior Perceived Physical Literacy Instrument

DSPG–*Dotazníku sebehodnocení pohybové gramotnosti*

CSPLQ—College Student Physical Literacy Questionnaire

The Perceived Physical Literacy Instrument (PPLI) [24] was the first physical literacy assessment validated for adults in 2016, adapted for Simple Chinese (PPLI-SC) in 2020 and Turkish in 2021, and recently in 2022 for the senior population (SPPLI). Both Simple Chinese and Turkish versions of PPLI, as well as SPPLI, employed factor analysis on the original pool of PPLI items for validation, resulting in adapted versions [30, 32, 48]. PPLI, and its adaptations, have been the most widely used instrument in included studies to assess physical literacy. All versions of PPLI reported satisfactory internal consistency and model fit. However, there was no evidence related to measurement invariance, concurrent validity, or temporal stability.

The College Student Physical Literacy Questionnaire (CSPLQ) was created specifically for young adults in 2022 [49]. Apart from internal consistency analysis, the authors performed concurrent validity testing with athletic ability, physical condition, physical attractiveness, physical fitness, frequency of physical activity, and length of physical activity variables.

*Dotazníku sebehodnocení pohybové gramotnosti* (DSPG) is a Czech instrument, created in 2019 and validated in young adults [34]. It was based on the Canadian physical literacy assessment PLAYself, originally developed for school-aged children [63]. DSPG was the only instrument to provide temporal stability evidence.

Ma and colleagues introduced a novel method to measure physical competence [50]. The study provided a database of speech designed to produce short-time automatic predictions of physical competence scores in CAPL2 physical literacy assessment. The method is based on the idea that there is a specific pattern of changes in an individual’s speech characteristics under different physical stresses [50]. In the future, speech analysis during exercise could become a valid method to predict physical competence as a part of physical literacy assessment. According to the authors, automated measurement tools can speed up assessments, thus saving time and improving accuracy.

A combined assessment approach was common in included studies. Four of them used a composite score, seven did not use it. Instruments and respective attributes are summarized in Table 6. Overall, diversity in assessments was observed. Practical tasks (ex. The Canadian Assessment of Physical Literacy, PLAYfun, The Test of Gross Motor Development 2, Timed Up and Go Test, Progressive Aerobic Cardiovascular Endurance Run), objective measures (ex. Body Mass Index, Waist circumference), and questionnaires (ex. The Physical Self-Perception Profile) were used to assess the physical domain. The behavioral domain was assessed by questionnaires (ex. International Physical Activity Questionnaire Short-Form, Physical Activity Stage of Change Assessment) and objective measures (ex. step counts, total physical activity tracking). The attitude domain was assessed by questionnaires (ex. The Behavioral Regulations in Exercise Questionnaire 3). The cognitive domain was assessed primarily by a self-constructed survey. This type of assessment method (self-constructed survey) was the most frequently employed (23% of studies), across all physical literacy domains.

**Table 6:** Instruments utilized in combined physical literacy assessments.

Instrument		Number of studies	Physical literacy attribute assessed
Questionnaire			
	BREQ-3 [64], BREQ3-PT [65]	5	Motivation
	MPAM-R [66]	1	Motivation
	SMS28 [67]	2	Motivation
	SIMS [68]	2	Motivation
	BREQ-2 [64]	2	Motivation
	Self-Efficacy for Exercise Questionnaire [69]	1	Confidence
	ECS [70]	1	Confidence
	ESES [71]	1	Self-confidence
	FKB-20 [72]	1	Self-confidence/self-efficacy
	RACK [73]	2	Self-efficacy
	Assessment of physical activity knowledge among US citizens [74]	1	Knowledge and understanding
	Outcome Expectations for Exercise Scale [69]	1	Understanding
	PNSE [75]	1	Motor competence
	PNTS [76]	1	Motor competence
	PSPP [77]	2	Perceived physical competence
	Physical activity attitudes scale [78]	1	Positive and negative physical activity attitudes
	Stanford Five City Study Questionnaire [79]	2	Attitude towards a physically active lifestyle
	IPAQ-SF [80]	4	Physical activity behaviors
	GPAQ [81]	1	Physical activity behaviors
	PSDQ-S [82]	1	Physical self-concept
	BAS-2 [83]	1	Body image
	PASCQ [84]	1	Physical activity state of change
	Eurobarometer questionnaire [85]	1	Opportunities
Practical task			
	Motor skill protocol [86]	1	Motor skill proficiency
	TGMD-2 [87]	1	Physical competence
	TUG [88]	1	Physical competence
	PLAYfun tool [89]	1	Movement competence
	PACER [90]	1	Physical fitness
	Grip strength	1	Physical fitness
	Sit-and-reach test	1	Physical fitness
	CAPL obstacle course [91]	1	Motor performance
Objective measure			
	Total physical activity tracking	2	Physical activity behaviors
	Step tracking	2	Physical activity behaviors
	BMI	1	Physical fitness
	Waist circumference	1	Physical fitness

IPAQ-SF—International Physical Activity Questionnaire Short-Form

PSPP—The Physical Self-Perception Profile

BREQ-3—The Behavioural Regulations in Exercise Questionnaire 3

TGMD-2—The Test of Gross Motor Development 2

BMI–Body Mass Index

TUG—Timed Up and Go Test

ECS—Exercise confidence survey

MPAM-R—Motives for physical activity measure-revised

PACER—Progressive Aerobic Cardiovascular Endurance Run

CAPL—The Canadian Assessment of Physical Literacy

ESES—Exercise Self-Efficacy Scale

SMS28—Sport Motivation Scale

SIMS—Situational Motivation Scale

FKB-20—Body Image Questionnaire

RACK—Risk Appraisal Consequences in Korea

GPAQ—Global Physical Activity Questionnaire

PSDQ-S—Physical Self-Concept Description Questionnaire Short-Form

BAS-2—Body Appreciation Scale-2

PNSE—Psychological need satisfaction in exercise scale

PNTS—Psychological Need Thwarting Scale

PASCQ—Physical Activity Stage of Change Assessment

## Discussion

This study aimed to systematically review existing physical literacy assessment attempts in adults. We found 31 studies, published from 2016 to 2022 and seven—from which only three were original—explicit physical literacy assessment instruments. Measurement properties were reported to a limited extent. The lack of available instruments led to a repeated utilization of self-constructed combined assessments. Most research was concentrated in China and published in the English language.

Since the completion of data collection for this review, additional physical literacy assessments have emerged, demonstrating the increasing research interest in this area. For instance, the Évaluation de la Littératie Physique [92]—an assessment instrument for emerging adults—has been developed, and a Persian version of the Perceived Physical Literacy Instrument has been validated in adults [93]. These recent developments highlight the need for ongoing review and synthesis of physical literacy assessment measures to keep up with the evolving field.

### Definition alignment and domains rationale

In “Physical literacy throughout the life course” Whitehead [2] describes physical literacy as an expression of fundamental capability based on monism philosophy: to develop physical literacy is to develop an embodied interaction with the world. Physical literacy domains—physical, cognitive, and affective—are highly interdependent, but, as stated by Whitehead, “monism does not prohibit attention being paid to the different dimensions that together comprise what it means to be human”. From this viewpoint, physical literacy assessment based on the assessment of discrete domains seems rational and practical. Essiet [94] suggested that tool developers may consider combining items from different scales and perform psychometric testing. Chen [95] criticized this approach and argued that it is impossible and makes no sense to break up physical literacy into independent components merely for measurement and maintain the monism premise.

While debate remains, studies included in the current review approached measurement in two different ways: by defining domains and assessing them discretely (through pre-validated or self-constructed instruments) and by defining domains as sub-scales after factorial analyses. The evolution of the Perceived Physical Literacy Instrument is a particularly interesting example of the latter. The original version of the instrument defined 3 domains: knowledge and understanding, self-expression and communication with others, sense of self and self-confidence [24]; the simple Chinese version defined other 3: motivation, confidence and physical competence, interaction with the environment [30]; the senior version yet another 3: attitude, ability, and sociality [48]. These versions were validated in different age groups: adults, young adults, and older adults. Does this discrepancy between domains indicate a need for varying physical literacy definitions, or just our inability to ask the right questions to assess physical literacy throughout life?

Studies that pre-defined domains faced another limitation, as the instruments they adopted were developed for an entirely different purpose rather than to measure physical literacy. For instance, motivation for engaging in sports activities measured by the Sport Motivation Scale and motivation to take part in physical activity for life are two completely different entities. The test of Gross Motor Development is a robust assessment of fundamental movement skills for children between the ages of 3 to 10 years and 11 months [96], but can it assess physical competence as a contextualized capability in adults, especially in older adults or people with disability? This common use of instruments borrowed from other contexts is an oversimplification of the concept and will not result in a better understanding of physical literacy.

In her work, Whitehead consistently discourages assessments that establish levels or comparisons with other individuals [2, 97, 98] and advocates for an assessment that allows charter individual progress and provides insight on possible improvement. However, no assessment included in the current review stated improvement purposes, and no study provided longitudinal data on physical literacy development with aging. Can an individual become more physically literate at an older age? Is it possible for a person who develops a disability to maintain physical literacy? Is assessment that creates feedback, which is then used to improve performance, a preferable approach to align with the physical literacy holistic definition? To answer these questions, we call for further qualitative and quantitative research that includes longitudinal data and compares different age groups, contexts, and assessment approaches.

### Assessment types

We found a fair use of both objective and subjective measures to assess different physical literacy attributes.

Objective measures, such as energy expenditure and step tracking, measure physical activity behavior and are easily obtained from wearable devices throughout the life course. In alignment with the physical literacy philosophical foundation, these measures embrace all movement and permit tracking individual’s progress. Ma [50] described a novel method to assess physical competence through speech evaluation. With an obvious potential to be integrated into a wearable device application, this kind of solution may become a future method of physical literacy assessment.

Practical tasks, frequently adapted from instruments developed for children, were used to assess motor performance and physical fitness. Assessed skills may not reflect a diversity of movement requirements during different stages of life [99], nor account for individual context. In the previous review by Edwards [11], most assessments in the physical domain evaluated physical competence, fundamental movement skills, and motor capacities in isolation instead of in applied settings. This consistency indicates a lack of progress in the understanding of what practical assessments could be applied conforming to physical literacy philosophy.

One of the premises that underpin the concept of physical literacy is based on an appreciation of the different modes through which the embodied dimension is lived [2]. The subjective perception of capabilities could be more valuable to describe one’s physical literacy and allows to avoid levels and standards. Studies included in the current review adopted pre-validated and self-constructed subjective measures to assess every domain. We found that self-constructed questionnaires were the most common assessment used, which signalizes an urgent need for further instrument development and validation. Self-perception, self-expression, and self-confidence—the key concepts of physical literacy as defined by Whitehead—were assessed through pre-validated scales. All explicit physical literacy assessment instruments were questionnaires (apart from Ma’s speech database which only assesses the physical domain). Despite the availability of questionnaire-based instruments for different physical literacy domains and attributes, it is not yet clear if such a complex holistic concept can be fully captured using exclusively subjective measures, or whether a combination of subjective and objective measures would be more appropriate.

### Usability and trustworthiness

We found that assessments’ measurement and usability characteristics were generally insufficiently reported. This lack of data makes it impossible to provide a comparison and make conclusions about the instruments’ quality and feasibility. We call for researchers to include all relevant information when creating, validating or applying physical literacy instruments. Reporting the time required for completion is essential, especially for assessments combining different scales and instruments. Practical tasks and objective measures are more time-consuming, and often require special equipment and trained staff. Studies included in the current review reported equipment and staff prerequisites of practical tasks, but only one reported the time needed to complete the assessment.

Poor measurement properties’ reporting can be partially explained by the common use of self-constructed scales. With a lack of invariance and temporal stability evidence even for validated instruments, it is unclear if those provide accurate results, especially over time.

We believe there is an opportunity for physical literacy researchers to create new instruments that could be reused and provide solid data. A recent review of physical literacy concept implementation in Europe demonstrated that the development of standardized assessment instruments may constitute an important step in intensifying physical literacy activities [100]. There is an urgent need for psychometric testing studies, that compare different assessments and re-test over time. Careful reporting of feasibility characteristics and providing information on the intended use of the instrument is essential to bring closer the universal adoption of the physical literacy concept.

### Public health agenda

We were not able to identify if any of the existing physical literacy assessments for adults is appropriate for large-scale/epidemiological studies. First, there is still no agreement between researchers on what constitutes the best way to capture such a complex multidimensional concept as physical literacy, or what attributes should be measured. Second, existing instruments lack validity and reliability data.

The first instrument validated in adults, Perceived Physical Literacy Instrument [24], has already been adapted for three languages and different populations, probably due to its simplicity and a one-fits-all approach. Further validation studies, especially longitudinal ones, are needed to understand if the Perceived Physical Literacy Instrument may be adopted for epidemiological studies and provide valuable insight into public health.

### Strengths and limitations

This systematic review on physical literacy assessments in adults provides valuable insights to the field. Firstly, the study offers a comprehensive review of existing assessment methods, providing an up-to-date and nuanced understanding of the available tools and their psychometric properties. This detailed analysis of assessment tools can help researchers, practitioners, and policy-makers make informed decisions about which tools are most appropriate for their specific contexts and purposes.

Additionally, the study provides a useful discussion of the definition and conceptualization of physical literacy, highlighting the importance of a holistic and context-dependent understanding of the construct. This discussion helps to clarify the meaning of physical literacy, its relevance to adult populations and can inform the development of more comprehensive and effective assessments.

Furthermore, the review contributes to the field by identifying gaps and limitations in current assessments of physical literacy in adults. For example, the study points out the need for further research on the psychometric properties and feasibility of different assessment instruments. Moreover, the study also identified a lack of progress in the understanding of how different types of assessments could provide better understanding of physical literacy. We suggest that a more comprehensive and integrated approach may be necessary. These insights can guide future research and development efforts to improve physical literacy assessments in adults.

To our best knowledge this is the first physical literacy assessment review to include studies in a non-English language. Apart from avoiding a language bias [18], we believe this inclusion is essential to provide information on how the physical literacy concept is being adopted around the world and how assessments are being culturally adapted. This can lead to more effective promotion of physical literacy concept and better outcomes for individuals and communities worldwide.

We adopted a holistic interpretation of physical literacy, which may have introduced a potential bias into our research. This bias stems from the fact that our analysis was grounded in a particular understanding of the concept of physical literacy, which may not be shared by all researchers in the field. Furthermore, our reliance on this holistic interpretation may have influenced our analysis of the data, potentially overlooking relevant aspects of physical literacy assessments that do not fit within this framework. We believe that in the importance of considering physical literacy as a multifaceted construct that goes beyond the simple measurement of different attributes and hope to inspire further research into the development of comprehensive and valid measures of physical literacy in adults.

## Supporting information

S1 File List of extracted items. (DOCX)

S2 File Raw data. (CSV)

S3 File Quality assessment summary. (CSV)

S1 Checklist Full PRISMA 2020 statement. (DOCX)
