# Supplementary material for: Physical literacy assessment in adults: A systematic review
Source: PLoS One. 2023 Jul 14;18(7):e0288541. doi: 10.1371/journal.pone.0288541 (PMC10348568; doi:10.1371/journal.pone.0288541)
Supplement: S1 File — (DOCX) [file pone.0288541.s001.docx]

**General information:**

1. Autor, year, and language
2. Publication type
3. Study design

**Content description:**

1. Age group (adults or older adults)
2. Country
3. Physical literacy definition adopted
4. Physical literacy domains assessed
5. Measurement used
6. Type of measurement
7. Setting
8. Strengths of assessment in relation to physical literacy (when specified)
9. Limitations of assessment in relation to physical literacy (when specified)

**Measurement properties:**

1. Sample size, gender, and age
2. Validity (type of validity, comparison measure, validity results when specified)
3. Reliability (time between test and retest and reliability results when specified)
4. Responsiveness (type of responsiveness, responsiveness results when specified)

**Assessment feasibility:**

1. Staff required (quantity and qualification)
2. Equipment required
3. Assessment time
4. Assessment administration format (online, on site, observation, questionary, practical task, etc.)
5. Adaptability
